# Evidence for Stabilizing Selection Driving Mutational Turnover of Short Motifs in the Eukaryotic Complementary Sex Determiner (Csd) Protein

**DOI:** 10.1534/g3.118.200527

**Published:** 2018-10-04

**Authors:** Vasco Koch, Marianne Otte, Martin Beye

**Affiliations:** Institute of Evolutionary Genetics, Heinrich Heine University Duesseldorf, Universitaetsstrasse 1, 40225 Duesseldorf, Germany

**Keywords:** mutational turnover, short linear motifs, post duplication divergence, protein evolution, complementary sex determiner (csd)

## Abstract

Short linear motifs (SLiMs) can play pivotal functional roles in proteins, such as targeting proteins to specific subcellular localizations, modulating the efficiency of translation and tagging proteins for degradation. Until recently we had little knowledge about SLiM evolution. Only a few amino acids in these motifs are functionally important, making them likely to evolve *ex nihilo* and suggesting that they can play key roles in protein evolution. Several reports now suggest that these motifs can appear and disappear while their function in the protein is preserved, a process sometimes referred to as “turnover”. However, there has been a lack of specific experiments to determine whether independently evolved motifs do indeed have the same function, which would conclusively determine whether the process of turnover actually occurs. In this study, we experimentally detected evidence for such a mutational turnover process for nuclear localization signals (NLS) during the post-duplication divergence of the Complementary sex determiner (Csd) and Feminizer (Fem) proteins in the honeybee (*Apis mellifera*) lineage. Experiments on the nuclear transport activity of protein segments and those of the most recent common ancestor (MRCA) sequences revealed that three new NLS motifs evolved in the Csd protein during the post-duplication divergence while other NLS motifs were lost that existed before duplication. A screen for essential and newly evolved amino acids revealed that new motifs in the Csd protein evolved by one or two missense mutations coding for lysine. Amino acids that were predating the duplication were also essential in the acquisition of the C1 motif suggesting that the *ex nihilo* origin was constrained by preexisting amino acids in the physical proximity. Our data support a model in which stabilizing selection maintains the constancy of nuclear transport function but allowed mutational turnover of the encoding NLS motifs.

Many studies have shown that protein domains cover only a fraction of a protein’s amino acid sequence, and functionally important short linear motifs (SLiMs) are often located in intrinsically unstructured regions (Björklund *et al.* 2005; Dyson and Wright 2005; Diella *et al.* 2008; Finn *et al.* 2016). These SLiMs are usually of low complexity, comprising just a few amino acids, and play pivotal functional roles such as controlling cell-cycle progression, tagging proteins for proteasomal degradation, modulating the efficiency of translation, targeting proteins to specific sub-cellular localizations (*e.g.*, nuclear localization signals) and stabilizing scaffolding complexes (Fuxreiter *et al.* 2007; Davey *et al.* 2012; Dinkel *et al.* 2014; Davey *et al.* 2015). To date, more than 200 motif classes have been curated using experimental validation (Dinkel *et al.* 2014). Until recently, little was known about SLiM evolution, especially in comparison to global domain evolution (for a review see (Davey *et al.* 2015)). The few amino acids that are functionally important make these domains very likely to arise *de novo* (*ex nihilo*) in a protein sequence through a small number of mutations (Neduva and Russell 2005; Davey *et al.* 2012). The potential for evolutionary changes in compact and degenerate SLiMs led to the hypothesis that they play a key role in protein evolution (Neduva and Russell 2005). Protein networks can acquire new interactions with only a few amino acid changes, thereby gaining important novel regulatory functions (Neduva and Russell 2005; Davey *et al.* 2015). There is accumulating evidence that new SLiMs can evolve *ex nihilo* (Davey *et al.* 2015). For example, several patients with the Noonan-like syndrome have independently evolved mutations in the lysine-rich repeat protein SHOC-2, which resulted in the *ex nihilo* birth of a myristoylation motif in humans (Cordeddu *et al.* 2009). Several analyses tracing the taxonomic range of motifs have shown that SLiMs are evolutionarily gained or lost in individual lineages. Extensive datasets provided by high throughput proteomics studies have shown that a large number of motifs are clade-specific (Holt *et al.* 2009; Goldman *et al.* 2014), suggesting that SLiMs have been repeatedly gained or lost. These gains and losses can be associated with functional changes of a protein. Many paralogous proteins gain distinct functionalities by gaining or losing SLiMs (Suijkerbuijk *et al.* 2012; Nguyen Ba *et al.* 2014; Di Fiore *et al.* 2015). For example, after the duplication of a Cyclin A/B ancestor, the Cyclin A regulatory subunit of the CDK protein kinase family gained an ABBA motif, allowing it to be degraded earlier than Cyclin B during prometaphase (Di Fiore *et al.* 2015). The *ex nihilo* birth of new motifs has also led to the hypothesis that motifs can appear or disappear while the protein retains its function, a process sometimes referred to as “turnover” (Moses and Landry 2010). Several reports have suggested that turnover might be a common mechanism in SLiM evolution. For example, many yeast cyclin-dependent kinase (Cdk) phosphorylation motifs are evolutionary transient, but the presence of a modification site(s) in a given protein region is conserved (Moses *et al.* 2007; Holt *et al.* 2009). However, specific experiments are needed to determine whether independently evolved motifs do indeed have the same function, to conclusively determine whether the process of turnover actually occurs (Moses and Landry 2010; Davey *et al.* 2015).

In this study, we experimentally found evidence for such a turnover process for nuclear localization signal (NLS) motifs during the post-duplication divergence of the Complementary sex determiner (Csd) and Feminizer (Fem) proteins in the honeybee lineage (*Apis mellifera*). We tested the amino acid sequences of a possible most recent common ancestor (MRCA) and demonstrated the gain of new NLS motifs by investigating the required amino acid changes.

Csd and Fem proteins are SR-type splice regulators that control sex determination via alternative splicing of the *fem* and *doublesex* (*dsx*) transcripts in the honeybee (*Apis mellifera*) (Beye *et al.* 2003; Gempe *et al.* 2009; Beye *et al.* 2013). The paralogous genes *csd* and *fem* evolved recently in the honeybee lineage by gene duplication (Beye *et al.* 2003; Hasselmann *et al.* 2008a; Hasselmann *et al.* 2010; Koch *et al.* 2014) (Figure 1), resulting in *csd* evolving as a new primary signal of sex determination in the honeybee (*Apis mellifera*). Meanwhile, the Transformer (Tra) proteins, which are orthologs of Fem, have retained their roles as splicing regulators (Hoshijima *et al.* 1991; Li and Bingham 1991; Pane *et al.* 2002; Hediger *et al.* 2010; Verhulst *et al.* 2010). Splice regulators need to be transported from the cytosol into the nucleus to perform the splicing process (though antibody staining of the location is lacking due to the absence of a specific antibody). This nuclear transport is controlled by NLS motifs that can vary in their amino acid composition (see (Fried and Kutay 2003; Yasuhara *et al.* 2009) for a review). Typical NLS motifs are dominated by basic amino acids that bind to importins, protein complexes that support the direct transport of proteins from the cell plasma to the nucleus through the nuclear pore (Fagerlund *et al.* 2002; Marfori *et al.* 2011). For example, bipartite NLS motifs consist of two clusters, each consisting of two to four basic amino acids (either lysine or arginine) separated by 10 amino acids (Dingwall and Laskey 1991; Robbins *et al.* 1991). A protein can carry several NLSs (Walther *et al.* 2005; Buck *et al.* 2006) that can be functional redundant (Südbeck and Scherer 1997; Parker *et al.* 2000). While individual NLSs are sufficient to promote nuclear transport of the protein, they can functionally be replaced by other NLSs present elsewhere in the protein.

**Figure 1:**
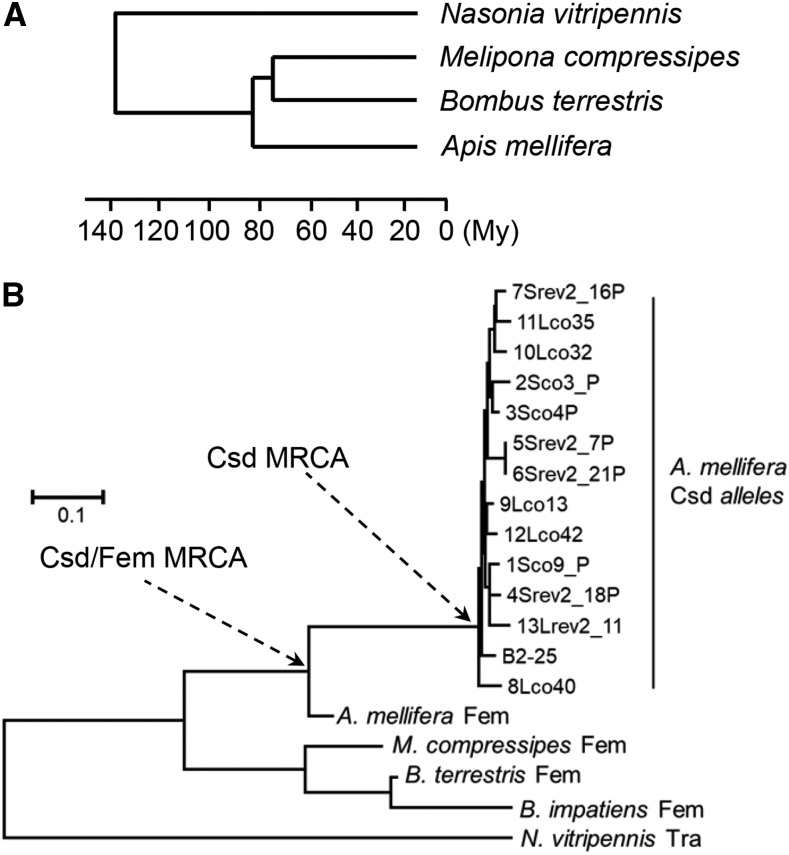
The evolutionary relationship of the bumble bee and honeybee species (*Apis mellifera*) and the post duplication divergence of the Fem and Csd proteins in the honeybee lineage (Hasselmann *et al.* 2008a). A: Time calibrated phylogenetic tree (in millions of years, My) of three bee species and one wasp species (Ramírez *et al.* 2010; Cardinal and Danforth 2013; Romiguier *et al.* 2016). B: The evolutionary history of protein sequences of selected bee and wasp species with the divergence of the Fem proteins and Csd alleles in the honeybee (*Apis mellifera*) lineage. The tree was inferred using the Neighbor-Joining method. Evolutionary distances were computed using the JTT matrix-based method and the modeling of rate variation among sites with a gamma distribution. The units are the number of amino acid substitutions per site. The species were: *Apis mellifera*, *Bombus terrestris*, *Bombus impatiens*, *Melipona compressipes*, *Nasonia vitripennis*. The Most Recent Common Ancestor (MRCA) of Csd and Fem proteins and Csd alleles in the honeybee lineage are marked with an arrow.

## Materials and Methods

### Cloning of nucleotide sequences

**Figure 2:**
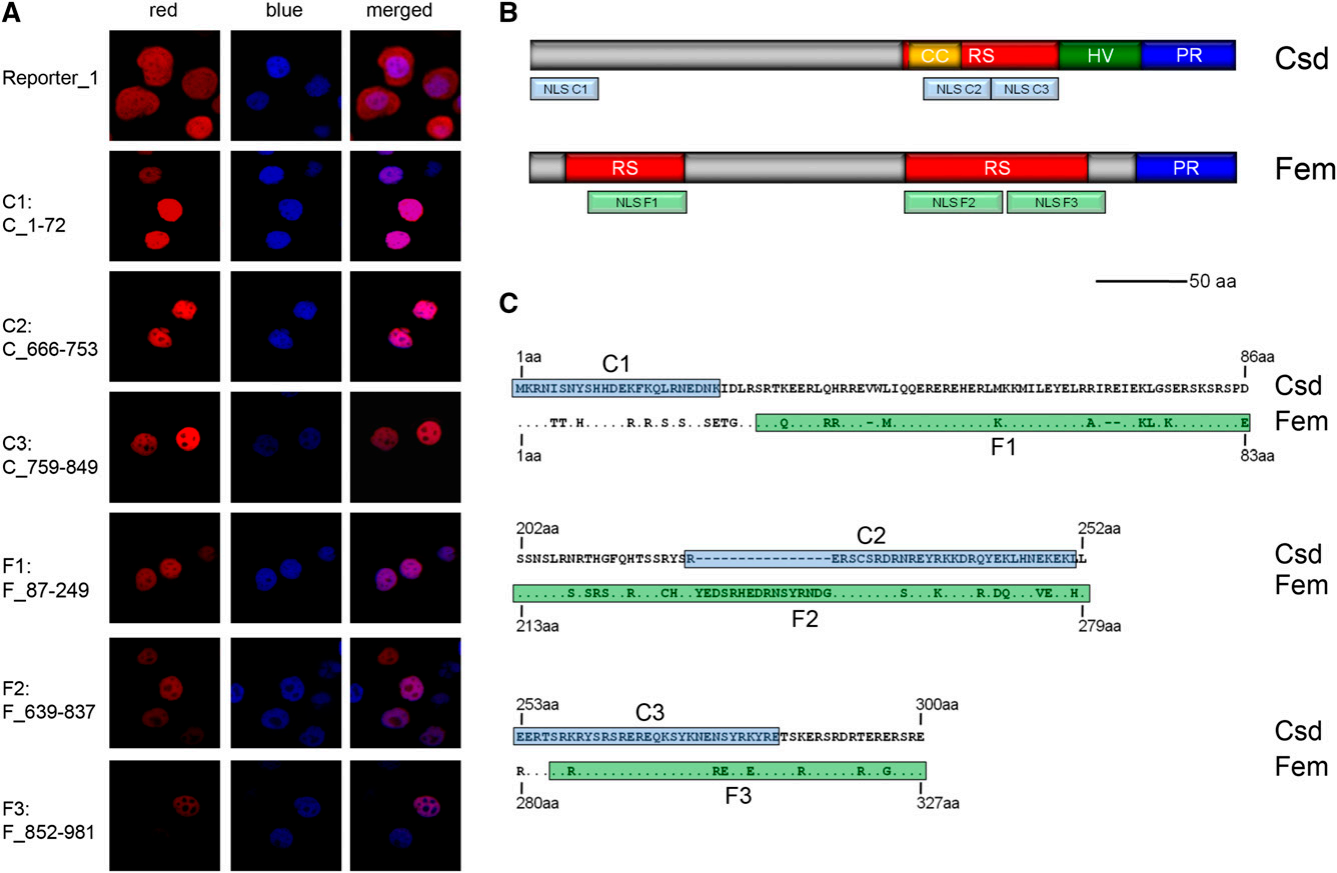
Minimal segments of the Csd and Fem proteins providing nuclear transport activity. A: The identified minimal segments of the Csd and Fem proteins that are sufficient to localize the reporter protein Rubia into the nucleus of *Sf*21 insect cells (red fluorescence signal in the pictures). The positive marker of nuclear localization (histone H2B fused with the Cerulean protein) was detected via its blue fluorescence signal. The nucleotide position encoding the tested segment of the allele *csd* B2–25 is shown to the left. The reporter protein without Csd- or Fem-derived segments is shown at the top (Reporter_1). Other segments examined are shown in Figs. S2 and S3. The different fusion proteins used are schematically presented in Fig. S1. B: The location of the segments providing nuclear localization function to the Csd and Fem proteins. The arginine-serine-rich region (RS domain), the coiled-coil motif (CC), the hypervariable region (HV region, confined to the Csd protein), and the proline-rich region (PR region) are shown. C: Amino acid sequence alignments of the minimal Csd and Fem segments. The C1 to C3 segments of the Csd protein are marked in light blue, while the F1 to F3 segments of the Fem protein are colored in light green.

**Figure 3:**
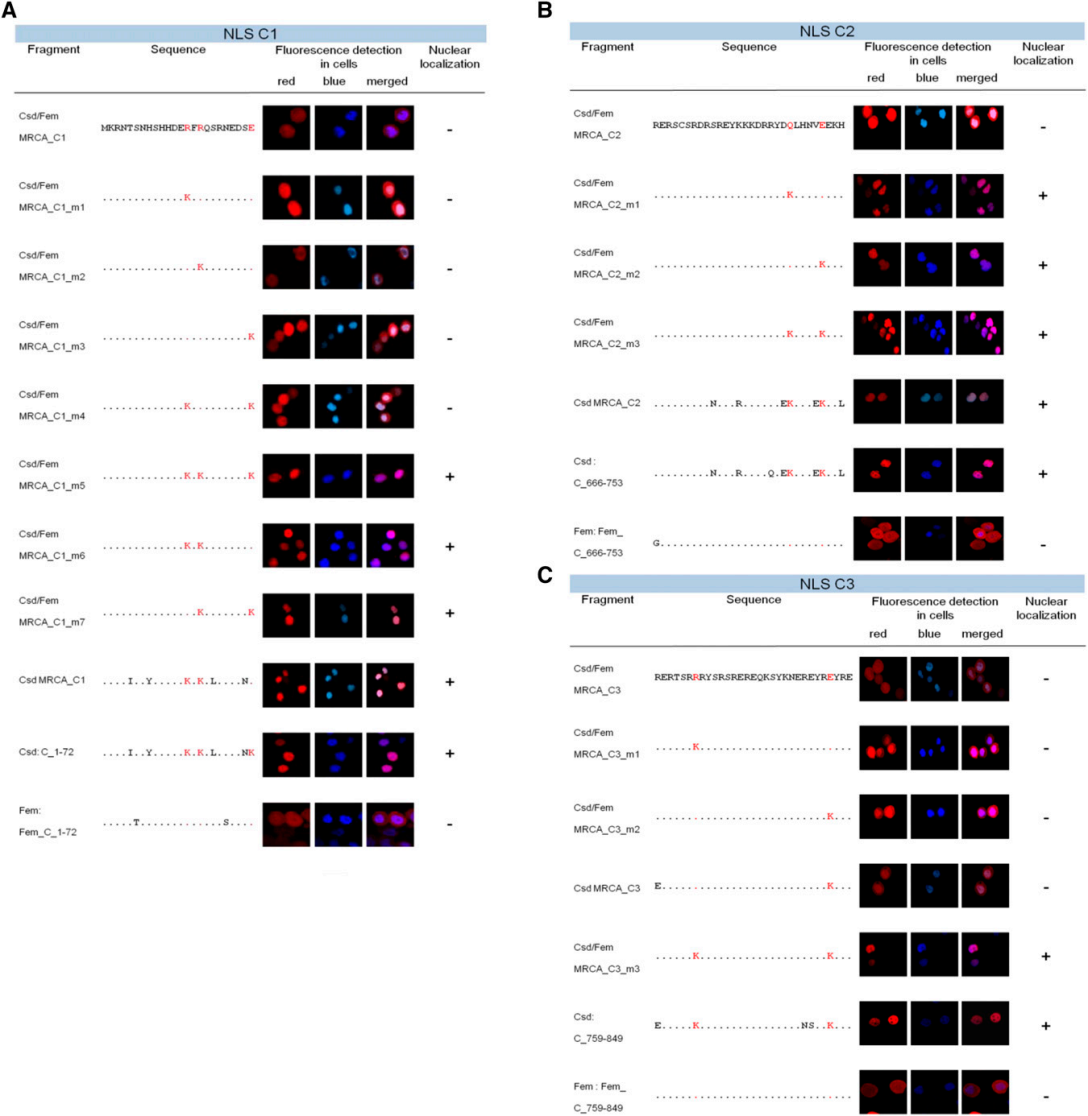
Evolutionary origin of NLS motifs in the C1, C2 and C3 segments through amino acid substitutions. A: Essential amino acid substitutions in the C1 segment for gaining nuclear localization activity through post-duplication divergence. B: Essential amino acid substitutions in the C2 segment for gaining nuclear localization activity through post-duplication divergence. C: Essential amino acid substitutions in the C3 segment for gaining nuclear localization activity through post-duplication divergence. The sites and the evolved amino acids in the Csd protein involved in the gain of nuclear transport function are shown in red letters. The sites are amino acid positions 14, 16 and 24 for the C1 segment; 243 and 248 for the C3 segment; and 259 and 280 for the C3 segment, with allele B2–25 as a reference. The red fluorescence signal in the pictures of *Sf*21 cells indicates the C-segment variants fused to the Rubia protein. The blue signal indicates the nuclear-localized H2B_Cerulean protein.

We first introduced Myc, Rubia and EGFP sequences into the PIZ/V5-His vector (Invitrogen, Carlsbad, CA, USA). The PIZ/V5-His vector was digested with *NotI* and *XbaI* to add a multiple cloning site, which was generated by polymerase chain reaction (PCR) using the oligonucleotide primers #27/#28 (Table S2). The resulting vector was digested using *XbaI* and *SacII*, and then the ORF of enhanced green fluorescent protein (EGFP) was amplified using oligonucleotide primers #01/#02 (Table S2) and inserted into the vector (Cormack *et al.* 1996). Subsequently, the ORF of the Rubia fluorescent protein (Schulte *et al.* 2013) was ligated into the pIZ/V5-His Spacer-EGFP plasmid using the restriction sites *EcoRI* and *NotI*. We also inserted a Myc tag and an *AflII* site using amplicons from oligonucleotide primers #37/#38 (Table S2), so the encoded proteins were fused with the N-terminus of the Rubia protein. The resulting plasmid (pIZ/V5-His Myc-*AflII*-Rubia-EGFP and Fig. S1) was used as a vector for the different *csd* and *fem* and derived sequences, as shown in Figures 2, 3, S2, S3, and S4, via *AflII* and *EcoRI* restriction sites. We also amplified the open reading frame (ORF) of Histone H2B from *Arabidopsis thaliana* using oligonucleotide primers #026/#027 (Table S2) and then inserted it into the pIZ/V5-His-Spacer-Cerulean plasmid via the *EcoRI* and *NotI* restriction sites. The pIZ/V5-His-Spacer-Cerulean plasmid was generated by cloning the ORF of the Cerulean fluorescent protein (Rizzo *et al.* 2004). For the analyses of the full-length Csd protein, we generated five mutational variants of the NLS sequences of *csd* allele B2–25. Briefly, we inserted the NLS sequences using the *AflII* and *EcoRI* restriction sites. The sequences with nucleotide changes were generated by PCRs with no template. The pIZ/V5-His-Csd MRCA of *csd* NLS 1-2-Rubia vector was generated in 3 steps. We amplified the *csd* B2–25 allele (#1a/#1d, Table S2) and inserted the amplicons into the pIZ/V5-His Myc-*AflII*-Rubia-EGFP vector via its *AflII* and *EcoRI* restriction sites. Next, via the *SapI* and *EcoRI* restriction sites, we inserted into this vector PCR amplicons generated with no template using oligonucleotide primers #2a/#2b (Table S2). In the last step, we introduced the amplicon of the *csd* B2–25 allele (#3a/#1c, Table S2) via *BbsI* and *EcoRI* restriction sites. The pIZ/V5-His-Csd NLS 1–3 mutated-Rubia vector was generated in 4 steps. We amplified the *csd* B2–25 allele (#1b/#1d, Table S2) and introduced the amplicon via the *AflII* and *EcoRI* restriction sites into the pIZ/V5-His Myc-*AflII*-Rubia-EGFP vector. In the same vector, we inserted the amplicons of oligonucleotide primers #2a/#2d (Table S2), generated with no template, via the *SapI* and *EcoRI* restriction sites. In the next step, we inserted a second amplicon (#3b/#3c, Table S2) generated with no template via the vector’s *BBSI* and *XhoI* restriction sites. In the last step, we inserted the amplicon of the *csd* B2–25 allele (#3d/#1c, Table S2) via *XhoI* and *EcoRI* restriction sites. To generate the pIZ/V5-His-Csd NLS 1 and 2 mutated-Rubia vector, we used the pIZ/V5-His vector, into which the amplicon of oligonucleotides #1b/#1d (Table S2) was already inserted. First, we inserted amplicons (oligonucleotide primers #2a/#2d and no template, Table S2) via the *SapI* and *EcoRI* restriction sites. Second, we inserted amplicons of the *csd* B2–25 allele (#3a/#1c, Table S2) via the *BbsI* and *EcoRI* restriction sites. We also generated pIZ/V5-His-Csd NLS 1 and 3 mutated-Rubia vectors. We used the pIZ/V5-His vector (which already possessed the amplicon from oligonucleotide primers #1b/#1d, Table S2). We inserted two PCR products that were generated without a template: the amplicon of #2a/#2b (Table S2) via the *SapI* and *EcoRI* restriction sites and the amplicon of #3b/#3c (Table S2) via the *BBSI* and *XhoI* restriction sites. In the last step, we inserted into the *XhoI* and *EcoRI* restriction sites the amplicon of the *csd* B2-25 allele generated with oligonucleotides #3d/#1c (Table S2). To construct the pIZ/V5-His-Csd NLS 2 and 3 mutated-Rubia vector we used a pIZ/V5-His vector that already possessed the amplicon of oligonucleotides #1a/#1d (Table S2). We introduced two amplicons generated with no template: the amplicon of #2a/#2d (Table S2) via the *SapI* and *EcoRI* restriction sites and the amplicon of #3b/#3c (Table S2) via the *BbsI* and *XhoI* restriction sites. Next, we inserted the amplicon of the *csd* B2-25 allele generated with #3d/#1c (Table S2) via the *XhoI* and *EcoRI* restriction sites. Since the expression of the Csd full-length protein from plasmids was very low and difficult to detect in *Sf*21 cells, we expressed the full-length Csd proteins using the baculovirus expression system (Invitrogen, Carlsbad, CA, USA). We cloned the *csd* sequences described above into pFastBac HTa vectors (Invitrogen, Carlsbad, CA, USA) using the restriction sites *MfeI* and *SalI*. Finally, we inserted each *csd* sequence in-frame with the Rubia fluorescent protein ORF (Fig. S1).

### Cell culture, transfection and microscopic analysis

*Sf*21 cells were adherently cultured in 10 ml of Spodopan medium (PAN-Biotech, Aidenbach, Germany) containing 10 μg/ml of gentamycin (Carl Roth, Karlsruhe, Germany) in 250 ml cell culture flasks (75 cm^2^; Greiner Bio-One). We transfected 1 × 10^6^ *Sf*21 cells with 2.5 µg of plasmid DNA via Roti-Insectofect reagent following the manufacturer’s instructions (Carl Roth, Karlsruhe, Germany). Baculovirus stocks were incubated following the Bac-to-Bac Baculovirus Expression System manual from Invitrogen (Invitrogen, Carlsbad, CA, USA).

We examined transfected *Sf*21 cells 24 to 72 hr after transfection using a confocal fluorescence microscope (Zeiss LSM510META, Carl Zeiss Microscopy, Jena, Germany) and an Achroplan 40×/0.8 W objective. Fluorescence was detected at wavelengths of 561 nm (Rubia) and 458 nm (Cerulean).

### Sequence analysis

We determined ancestral sequences (Fig. S7) using the ANC-GENE software (Zhang and Nei 1997) and the MEGA5 software package (Tamura *et al.* 2011) using nucleotide sequence alignments. We used the distance-based Bayesian method to infer the ancestral amino acid states and then inferred the underlying nucleotide sequences by fixing the inferred amino acids. ANC-GENE and MEGA5 yielded identical results for the ancestral amino acid states of the NLS elements. The following *csd*, *fem* and *tra* sequences were used for the analysis (GenBank accession numbers): *Apis mellifera csd* alleles: B2-25 (AY569703.1); Sco9_P (EU100895.1); Sco3_P (EU100893.1); Sco4P (EU100894.1); Srev2_18P (EU100898.1); Srev2_7P (EU100896.1); Srev2_21P (EU100896.1); Srev2_16P (EU100897.1); Lco40, (EU100888.1); Lco13 (EU100885.1); Lco32 (EU100886.1); Lco35 (EU100887.1); Lco42 (EU100889.1); Lrev2_11 (EU100890.1). *Apis mellifera fem* (AY569719.1); *Bombus terrestris fem*: (100628566); *Bombus impatiens* (100742483) *Nasonia vitripennis transformer* (*tra*)(EU780924.1); *Melipona compressipes*; *fem* (EU139305.1). To test whether the similarity between the sequences was sufficiently high to generate informative MRCA nucleotide and amino acid sequences (Table S1), we performed tests on the saturation of substitutions at the 3^rd^ codon position between *Bombus* and *Apis* sequences using the methods implemented in the DAMBE6 program (Xia *et al.* 2003; Xia 2017) and examined the number of synonymous substitutions per synonymous site between nucleotide sequences (*ds*) using the Nei-Gojobori model, which is implemented in the MEGA5 software package (Tamura *et al.* 2011). We used NLS mappers to identify possible NLSs in the Csd amino acid sequence (http://nls-mapper.iab.keio.ac.jp/cgi-bin/NLS_Mapper_form.cgi (Kosugi *et al.* 2009); http://www.moseslab.csb.utoronto.ca/NLStradamus/ (Nguyen Ba *et al.* 2009), last successful access October 2017). and IUPred2A program to identify disordered regions (Mészáros *et al.* 2018) (https://iupred2a.elte.hu/ last successful access August 2018). The evolutionary history of the Fem and Csd protein sequences were inferred with models implemented in the MEGA5 software package (Tamura *et al.* 2011). We used Neighbor-Joining method with the tree drawn to scale, with branch lengths in the same units as those of the evolutionary distances. Maximum likelihood fits were run to identify best fit amino acid substitution models. Evolutionary distances were computed using the JTT matrix-based method and the modeling of rate variation among sites with a gamma distribution (shape parameter = 1). All positions containing gaps and missing data were eliminated.

### Data Availability Statement

Plasmids are available upon request. The authors affirm that all data necessary for confirming the conclusions of the article are present within the article, figures and supplementary data set available at Figshare: https://figshare.com/s/944fea8a9dfa2045b1f8. 

## Results

### New NLS motifs evolved in the Csd protein

To investigate the evolution of their localization function post divergence, we characterized minimal segments of the Fem and Csd proteins along with MRCA sequences and tested whether they were sufficient to direct localization into the nucleus. The sequences in this study were fused to the red fluorescent protein Rubia with spacer sequences, and then they were expressed in *Sf*21 insect cells using expression or baculovirus systems (Figure 2). We tested three minimal segments for the Csd protein (C1: aa 1–24, 24 aa long; C2: aa 222–251, 30 aa long; C3: aa 253–283, 31 aa long) and three for the Fem protein (F1: aa 29–83, 55 aa; F2: aa 213–279, 67 aa; F3: aa 284–327, 44 aa) that were sufficient for nuclear localization (Figures 2, S2 and S3). We used the Histone H2B protein from *Arabidopsis thaliana* as a marker of nuclear localization because histone proteins are typical proteins that need to be transported into the nucleus. Each of these constructs co-localized with the Histone H2B protein from *Arabidopsis thaliana*, fused with the blue fluorescent protein Cerulean, which we used as a positive marker for nuclear transport (Figures 2 and S1). As a control, the reporter protein was expressed alone (Figures 2 and S1), and it was not transported into the nucleus. When we further reduced the size of the Csd and Fem segments, the proteins were not transported to the nucleus but were instead detected in the cytoplasm (Figs. S2 and S3). These segments are all located in disordered domains of the proteins consistent with the location of short motifs in other proteins (Björklund *et al.* 2005; Dyson and Wright 2005; Diella *et al.* 2008; Finn *et al.* 2016). Next, we inferred the sequence of the Csd and Fem MRCA (Csd/Fem MRCA, Figure 1B) and that of Csd (Csd MRCA) by applying a Bayesian approach to alignments of the coding sequences comprising multiple *csd* alleles and *fem* sequences from the honeybee and *fem* sequences from three other bee species and the ortholog *tra* sequence from the wasp *Nasonia* (Figure 1; Table S1). MRCA sequences of the Csd/Fem C1, C2 and C3 segments, representing the ancestral state prior to the duplication and divergence of Csd and Fem proteins, were not transported to the nucleus (Csd/Fem MRCA_C1, _C2 and _C3; Figure 3 A, B and C). The C1, C2 and C3 homologous segments of the Fem protein (Fem_C_1–72, Fem_C_666–753 and Fem_C_759–849) were also not sufficient to direct nuclear localization (Figure 3). These results together indicate that three NLS motifs evolved during the post-duplication divergence of the Csd protein. Further, we examined whether this divergence occurred in the branch before or in the branches after the divergence into multiple Csd alleles (Figure 1B). This examination is possible because Csd alleles have been maintained by strong balancing selection over millions of years (Hasselmann *et al.* 2008b; Lechner *et al.* 2014). To interrogate this, we examined the nuclear transport of the MRCA sequences of the Csd alleles (Csd MRCA, Figure 1B) in the C1, C2 and C3 segments. We observed that Csd MRCA_C1, Csd MRCA_C2 and Csd MRCA_C3 were transported into nucleus (Figure 3). Together, these results indicate that C1, C2 and C3 NLS motifs evolved after duplication, but prior to the divergence of different Csd alleles. As for the F elements of the Fem protein, we were only able to infer the Csd/Fem MRCA sequence of the F1 segment. The Csd/Fem MRCA_F1 sequence was transported into the nucleus (Fig. S4), indicating that this transport function is conserved and pre-dated the duplication and divergence of the Csd and Fem proteins. Independent deletions and insertions of the homologous F2 and F3 segments in different bee lineages made it impossible to infer the F2 and F3 Csd/Fem MRCA sequences. To further explore the history of the F2 and F3 segments, we examined the transport function of the homologous F2 and F3 segments of the bumblebee *Bombus terrestris fem* gene. The F3 segment of *B. terrestris* was transported into the nucleus, while the F2 element was not (Fig. S4), suggesting that the transport function of the F3 segment also pre-dated the duplication event.

### Nuclear transport functions repeatedly evolved via one or two missense mutations coding for lysine

Next, we screened for amino acids that evolved after duplication in the Csd protein resulting in the gain of nuclear transport. We introduced amino acids into the Csd/Fem MRCA sequence that evolved during the post-duplication divergence, but prior to the divergence of the Csd alleles (*i.e.*, changes detected between the Csd/Fem MRCA and Csd MRCA sequences) and tested for nuclear transport. To understand the evolution of the elements also after allele divergence we introduced those amino acids that evolved in lineage of the allele CsdB2-25 (Figures 3, S5).

For the C1 segment, we observed that two post-duplication changes (replacement of R^14^ (arginine) with K^14^ (lysine) and R^16^ with K^16^) were sufficient to direct nuclear transport (Figure 3A). The E^24^ (glutamic acid) to K^24^ change that evolved after the divergence of Csd alleles was also sufficient to mediate nuclear transport, but only together with the R^16^ to K^16^ replacement. Nucleotide sequence analysis (Table 1) revealed that each of the amino acid changes resulted from single nonsynonymous nucleotide replacements. Together, these results suggest that a new NLS motif in C1 evolved during the post-duplication divergence of the Csd and Fem genes by two missense mutations coding for the amino acid lysine.

**Table 1:** The amino acid (aa) and nucleotide states at the sites of the functionally relevant lysines before the Csd/Fem duplication and divergence event (Csd/Fem MRCA) and before the Csd allele divergence (Csd MRCA)

		aa/codon of Csd B2–25	aa/codon of Csd/Fem MRCA (*P* > 0.9)	aa/codon of Csd MRCA (*P* > 0.9)	aa/codon polymorphisms (frequency %)^3)^
NLS	C1	K^14^	R	K	K (100%)
		AAA	AGA	AAA	AAA
		K^16^	R	K	K (100%)
		AAA	AGA	AAA	AAA
		K^24^	E	E	E (57%) K (43%)
		AAA	GAA	GAA	GAA AAA
	C2	K^243^	Q	K	K (100%)
		AAA	CAA	AAA	AAA
		K^248^	E	K	E (7%) K (93%)
		AAA	GAA	AAA	GAA AAA
	C3	K^259^	R	K	E (14%) K (79%) N (7%) GAG AAG AAC
		AAG	AGG	AAG^2^)	
		K^280^	E ^1)^	K	K (21%) K (79%)
		AAG	GAA	AAA	AAG AAA

1) Ambiguous codon (*P* < 0.9) due to indels that occurred with outgroup sequence comparison.

2) The predicted codon was R (AGG), *P* < 0.6 using ANC-GENE (Zhang and Nei 1997), and K (AAG), *P* > 0.9 using MEGA (Tamura *et al.* 2011). From the more parsimonious number of mutations required to produce the other polymorphism (GAG and AAC), we suggest that the aa/codon of the *csd* MRCA is K/AAG.

3) Estimated from a random sample of 14 *csd* alleles.

For the C2 segment, we discovered that replacing either Q^243^ (glutamine) with K^243^ or E^248^ with K^248^ in the Csd/Fem MRCA_C2 sequence was sufficient to mediate nuclear localization (Figure 3B). Both lysines evolved by single nucleotide changes (Table 1). K^243^ evolved post duplication while K^248^ either evolved post duplication or post divergence of the Csd alleles (Table 1). These results suggest that a new NLS motif in the C2 segment evolved by a single missense mutation coding for lysine.

For the C3 segment, we found that substituting R^259^ with K^259^ and E^280^ with K^280^ in the Csd/Fem MRCA_C3 sequence was sufficient to direct nuclear localization (Figure 3C). Both lysine-encoding codons evolved by single nucleotide changes (Table 1). K^259^ and K^280^ (Table 1) evolved post duplication. Together, these results indicate that the new NLS motif in the C3 segment evolved by two single missense mutations coding for lysine.

### The evolution of the NLS motif in C1 was constrained by preexisting amino acids

Our results indicate that new NLS motifs can evolve via one or two missense mutations. We next investigated whether there are constraints on the pre-existing sequence on where in a protein the new motifs can evolve. To address this question, we used a C1 Csd/Fem MRCA sequence with the lysine changes K^14^ and K^16^. This sequence represented the ancestral sequence (prior to duplication) with the two mutations that gained the new nuclear transport function (Fig. S7). We then replaced amino acids that pre-existed prior to duplication and tested whether these ancestral amino acids were essential for the gain of nuclear transport together with the newly evolved lysine. Replacing basic amino acids lysine (K) or arginine (R) at sites 2, 3 and 19 with either of the neutral amino acids alanine or glycine resulted in loss of nuclear transport function (Fig. S7). Substituting glutamic acid (E) at site 13 with alanine or glycine had no such effect (Fig. S7). These observations indicate that several basic amino acids existed prior to duplication that became essential for the rise of the new NLS motif. This result indicates that the origin of a new motif by two mutations also required other basic amino acids in close proximity that pre-existed in the ancestral sequence.

### Newly evolved lysines were essential in producing three NLS motifs in the Csd protein

We next investigated whether the newly evolved NLS motifs in the C1, C2 and C3 segments were indeed active in the full-length Csd protein. To investigate this, we mutated the newly evolved lysines in each motif and examined the nuclear transport of the entire protein.

**Figure 4:**
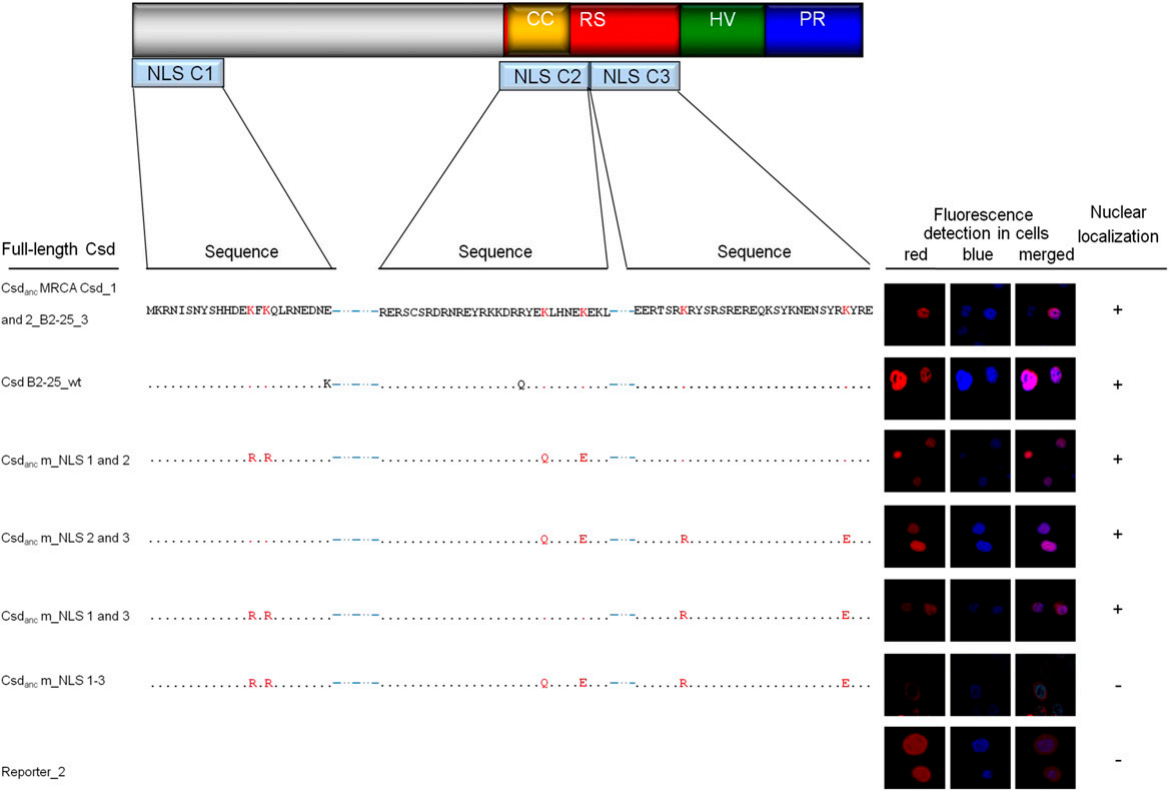
Essential role of the newly evolved lysines in the transport of the Csd protein. A: Schematic view of the domain structure of the Csd protein with the relative positions of the NLS elements. The arginine-serine-rich region (RS domain), the coiled-coil motif (CC), the hypervariable region (HV region), and the proline-rich region (PR region) are shown. B: Sequence changes in the C1, C2 and C3 segments of the Csd protein and their consequences on nuclear transport. The newly evolved lysines that gave rise to the NLS function, as determined in the segment analysis, were reverted to the ancestral state (Csd/Fem MRCA sequence) in each C segment. The sites that were changed are colored in red (codon sites 14, 16 for C1; 243 and 248 for C2; and 259 and 280 for C3). The red fluorescence signal in the pictures of *Sf*21 cells indicates the Csd protein variants fused to the Rubia protein. The blue signal represents nuclear H2B_Cerulean protein marking the nucleus. The images behind each sequence show the localization of that protein when expressed in *Sf*21 cells. The proteins (Fig. S1D) were fused with the Rubia protein and detected via red fluorescence. The nucleus was detected as the blue fluorescence of the histone H2B-Cerulean protein. Reporter_2 is the reporter protein not fused with Csd (fig. S1E).

First, we generated and expressed the Csd _MRCA Csd_1 and 2_ sequences from Csd allele B2–25. This protein contained the amino acid sequences at the C1, C2 and C3 segments from before the Csd allele divergence. We observed that the Csd _MRCA Csd_1 and 2_ proteins were transported into the nucleus (Figure 4), suggesting that the transport function was present prior to the Csd allele divergence. Next, we expressed the Csd _m_NLS1-3_ sequence, in which all six functionally relevant lysines of all three segments (K^14^ and K^16^ for C1; K^243^ and K^248^ for C2; and K^259^ and K^280^ for C3) were reverted to their ancestral state prior to duplication. This Csd _m_NLS1-3_ sequence was not transported to the nucleus (Figure 4) suggesting that no other NLS motifs are present in the Csd protein. Next, we reintroduced, stepwise for each motif, the evolved lysines into Csd _m_NLS1-3_ and tested for gain of transport function. Introducing lysines (K^14^ and K^16^) of C1 into the Csd _m_NLS1-3_ protein resulted in nuclear transport (Csd _m_NLS 2 and 3_; Figure 4) indicating that the evolution of those two lysines was sufficient to mediate nuclear transport of the Csd protein. Introducing either lysines K^243^ and K^248^ of C2 or lysine K^259^ and K^280^ of C3 into the Csd _m_NLS1-3_ protein also mediated nuclear transport (Csd _m_NLS 1 and 3_, Csd _m_NLS1 and 2_; Figure 4), suggesting that the evolved lysines in the C2 or C3 motifs are each sufficient to mediate the nuclear transport of the Csd protein. Together our results indicate that each of the three newly evolved motifs can mediate nuclear transport of the Csd protein.

## Discussion

Our results provide experimental evidence for the mutational turnover of NLS motifs after the divergence of the Csd and Fem proteins. Three NLS motifs newly appeared in the Csd proteins by one or two point mutations, while the evolutionarily older motifs that existed in the common ancestor of the Csd/Fem proteins were lost, demonstrating motif turnover and the preservation of nuclear transport. Several reports have suggested that turnover might be a common mechanism in SLiM evolution (Moses and Landry 2010; Davey *et al.* 2015). For example, studies on yeast cyclin-dependent kinase (Cdk) have identified specific phosphorylation motifs that have changed, while the presence of modification sites in given protein regions has been conserved (Moses *et al.* 2007; Holt *et al.* 2009). However, with data presented here we have demonstrated that newly evolved motifs can indeed have the same function as their ancestral sequence, which provides experimental support for the turnover model.

Our data support a model in which stabilizing selection maintains the constancy of nuclear transport function but allowed mutational turnover of the encoding NLS motifs. One driving force for this turnover of motifs is the ease by which new motifs can evolve *ex nihilo* through a small number of mutations (Lynch 2007). Random genetic drift or further adaptive adjustment and selection for new functions are possible evolutionary forces that may drive new variants in the population to fixation. Interestingly, turnover due to stabilizing selection at the level of gene regulation is a common model for the evolution of transcription factor binding sites *in cis*-regulatory modules. There is strong support from the results of genome-wide and single gene-based studies (Ludwig *et al.* 2000; Dermitzakis and Clark 2002; Moses *et al.* 2006; Bradley *et al.* 2010; Arnold *et al.* 2014). For example, despite high sequence divergence, the *eve* stripe enhancer regions from closely related species drive nearly indistinguishable expression patterns in *Drosophila melanogaster* (Ludwig *et al.* 1998), while the specific transcription factor binding sites responsible for their expression patterns seem to have changed during evolution (Ludwig *et al.* 2000). Our results on NLS motifs suggest a related phenomenon of stabilizing selection for the evolutionary turnover of protein SLiMs.

Our results on the mutational steps essential for the creation of three NLS motifs further support the model of *ex nihilo* SLiM evolution by a small number of mutations (Neduva and Russell 2005; Davey *et al.* 2015). Only two replacements in the C1 and C3 and one replacement in C2 segment, all with the amino acid lysine, were sufficient to give birth to new NLS motifs in the Csd protein. These changes required only single nonsynonymous mutations, suggesting that new motifs may indeed arise by chance (Davey *et al.* 2015). Further, for the C1 and C3 motifs a single mutation alone was not sufficient to direct even slight nuclear transportation, indicating that partial gain of function was not driving motif acquisition. We also revealed that amino acids that were predating the duplication were essential in the acquisition of the C1 motif suggesting that the *ex nihilo* origin of SLiMs is constrained by preexisting amino acids in the physical proximity.

The three newly evolved NLS motifs were functionally redundant in our transport assay, suggesting that all of them are functionally relevant in honeybees. This finding is consistent with reports of other protein families having multiple NLSs (Walther *et al.* 2005; Buck *et al.* 2006); other members of the protein family from the present study are all splice regulators that are transported into the nucleus (Hoshijima *et al.* 1991; Li and Bingham 1991; Pane *et al.* 2002; Hediger *et al.* 2010; Verhulst *et al.* 2010). Neither our algorithmic nor our data bank-based motif analyses have predicted the identified NLS motifs. However, the pattern of the essential amino acid lysine to the left and right of the minimal segments in all three motifs suggest that the newly evolved motifs belong to the class of bipartite NLSs (Dingwall and Laskey 1991; Makkerh *et al.* 1996).
